# LUNG group 2 innate lymphoid cells as a new adjuvant target to enhance intranasal vaccine efficacy against influenza

**DOI:** 10.1002/cti2.1381

**Published:** 2022-03-27

**Authors:** Clare M Williams, Sreeja Roy, Wei Sun, Andrea M Furuya, Danushka K Wijesundara, Yoichi Furuya

**Affiliations:** ^1^ Department of Immunology and Microbial Disease Albany Medical College Albany NY USA; ^2^ Pictor Limited Auckland New Zealand; ^3^ The School of Chemistry and Molecular Biosciences The Australian Institute for Bioengineering and Nanotechnology The University of Queensland Brisbane QLD Australia

**Keywords:** IL‐33, influenza, lung group 2 innate lymphoid cells, mucosal vaccination, Th2

## Abstract

Group 2 innate lymphoid cells (ILC2) are a relatively new class of innate immune cells. Lung ILC2 are early responders that secrete type 2 cytokines in response to danger ‘alarmin’ signals such as interleukin (IL)‐33 and thymic stromal lymphopoietin. Being an early source of type 2 cytokines, ILC2 are a critical regulator of type 2 immune cells of both innate and adaptive immune responses. The immune regulatory functions of ILC2 were mostly investigated in diseases where T helper 2 inflammation predominates. However, in recent years, it has been appreciated that the role of ILC2 extends to other pathological conditions such as cancer and viral infections. In this review, we will focus on the potential role of lung ILC2 in the induction of mucosal immunity against influenza virus infection and discuss the potential utility of ILC2 as a target for mucosal vaccination.

## Graphical abstract

Group 2 innate lymphoid cells (ILC2) are a relatively new class of innate immune cells that play pivotal roles in orchestrating protection against respiratory mucosal pathogens. In this review, we focus on the potential role of lung ILC2s in induction of mucosal immunity against influenza virus and discuss the potential utility of ILC2s as a target for mucosal vaccination.

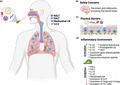

## Introduction

Current influenza vaccines are developed by predicting influenza viruses with the potential to circulate in the forthcoming seasons. Seasonal influenza vaccines in general provide immunity against contemporary viral strains in circulation with limited cross‐protection against drifted variants. These vaccines commonly rely on eliciting a neutralising antibody response against the highly variable regions of surface antigens such as hemagglutinin (HA) and mutations in HA can lead to escape variants that result in the reduction of vaccine efficacy.^1^ Thus, there is an urgent need to develop influenza vaccines that elicit broad and long‐lasting immunity against conserved regions of the viral proteome to prevent future influenza epidemics and/or pandemics. To this end, targeting the nucleoprotein (NP), matrix protein and the stem region of HA has been shown to confer cross‐protective immunity against heterologous influenza viruses in animal models.^1^

It is generally accepted that type 1 immunity orchestrated T cells, neutralising antibodies and non‐neutralising antibodies that mediate cytotoxicity of infected cells are required for optimal protection against influenza. In this regard, the cross‐protective role of cytotoxic T cells is well established, particularly for heterologous strains for which responses against conserved epitopes are important. In contrast, the induction of type 2 immune responses during a viral infection is often considered pathological. Adoptive transfer studies evaluating the protective role of type 1 helper T (Th1) cells vs. type 2 helper T (Th2) cells against influenza virus infection have demonstrated that cytolytic Th1 cells are protective, but not type 2 cytokine expressing Th2 cells.^2^ Th2 cells not only worsened the lung immunopathology but also delayed influenza viral clearance.^2^ In a more recent study, we reported a contradictory role of type 2 immune responses during influenza infection. Specifically, interferon (IFN)‐γ deficient mice exhibited increased resistance against primary influenza virus infection, which was dependent on the group 2 innate lymphoid cell (ILC2) response.^3^ While these studies shed light on the role of type 1 vs. 2 immunity during primary influenza virus infection, their relative contribution during subsequent heterologous infections, which commonly occurs in humans, is not fully understood. In particular, due to the known association between immunopathology and dysregulated type 2 immune responses during viral infections, the potential contribution of type 2 immunity in cross‐protection against influenza virus following infection and mucosal vaccination has been understudied and unexplored.

## Importance of intranasal vaccination for protection against respiratory virus infection

It is well established that mucosal or local deposition of the vaccine at the site of pathogen exposure is the most effective vaccination strategy to elicit sterilising immunity and protection against mucosal pathogens.^4^ Consequently, despite the challenges in the human vaccination context, intranasal (i.n.) vaccination strategies are being pursued to develop dose‐sparing and efficacious vaccines against respiratory viruses with pandemic potential such as influenza virus and severe acute respiratory syndrome coronavirus 2 (SARS‐CoV‐2). As an example, i.n. vaccination using a single dose of the licenced Chimpanzee Adenovirus platform encoding SARS‐CoV‐2 Spike (ChAd‐SARS‐CoV‐2‐S) provided superior immunity and protection against SARS‐CoV‐2 in the upper and lower respiratory tract compared to a single dose, intramuscular (i.m.) vaccination with the same vaccine.^5^ In this study, a 2‐dose prime‐boost vaccination regimen was required to elicit protection following i.m. vaccination, which further demonstrates the importance of local i.n. vaccine delivery.^5^ Influenza vaccine studies have also shown that the i.n. route is more effective in eliciting protection than the i.m. vaccination in a dose‐sparing manner. Notably, parenteral routes of vaccine delivery are less effective in eliciting IgA responses in the upper respiratory tract.^4^, ^6^

Currently, there is no licenced i.n. vaccine based on inactivated antigens. This is largely due to low immunogenicity of inactivated antigens at mucosal surfaces, requiring effective mucosal adjuvants in order to elicit optimal responses. Indeed, the first licenced inactivated i.n. influenza vaccine in the world for the 2000–01 influenza season was adjuvanted with heat‐labile *Escherichia coli* enterotoxin. However, this vaccine was withdrawn from the market due to a strong association between the i.n. vaccination and Bell’s palsy.^7^ The mechanism behind induction of Bell’s palsy is still not clear, but it is believed that the interaction between enterotoxin and ganglioside GM1, a high‐affinity receptor for enterotoxin, on nerve cells played a role. Thus, the most significant challenge with i.n. vaccination is to ensure that the vaccine formulation does not traverse the blood brain barrier and is safe. The use of reporter systems (e.g. reporter viruses) have allowed researchers to track recombinant vaccines to ensure that they are delivered in a targeted manner, but in the case of vaccines for which this is not possible (e.g. subunit vaccines requiring the use of adjuvants), accommodating i.n. deliverable vaccines are challenging. From our own experience, manufacturers of adjuvants established for i.m. delivery are reluctant to test i.n. delivery modalities, especially in current times, to avoid controversies fuelled by the anti‐vaxxer movements, which is a significant concern in the COVID‐19 pandemic era for public confidence in vaccination. The spike subunit vaccine developed recently, SARS‐CoV‐2 Sclamp, which exhibited a robust safety and immunogenicity profile in a double‐blinded, randomised Phase I clinical trial was not advanced to Phase II because the stabilisation domain of the trimeric spike was derived from the HIV‐1 glycoprotein 41, which was immunogenic and resulted in a diagnostic interference with some HIV diagnostic assays.^8^, ^9^ Although none of the trial participants were HIV positive, the Australian government feared issues with vaccine confidence in the general public and stopped this trial. Despite these caveats and the modest efficacy of i.n. FluMist vaccine delivery in humans, a recent review has highlighted strategies in terms of antigen choice, adjuvants and delivery, among other factors, which can be optimised to potentially lead to the development of effective i.n. vaccines for use in humans.^4^ Given that the contribution of ILC2 in vaccine responses is underappreciated, the primary focus of this review is to discuss the potential utility of targeting ILC2 in the respiratory mucosa and lungs following mucosal (i.n.) vaccination.

## Type 2 immunity and influenza virus infection

Acute pulmonary viral infections such as influenza virus infection have typically been considered to elicit a type 1 biased immune response; however, the abundance of type 2 cytokines is also detected during influenza virus infection. Early studies have demonstrated the protective role of type 2 cytokine secreting CD8^+^ T cells. *In vitro* generated and adoptively transferred influenza virus hemagglutinin (HA)‐specific type 2 CD8^+^ T that expressed interleukin (IL)‐4, IL‐5, IL‐10 and IL‐13 cells protected recipients following PR8 influenza virus challenge, although the protection was greater in HA‐specific type 1 CD8^+^ T cell recipients.^10^ Of note, due to IL‐5 production, type 2 CD8^+^ T cells promoted pulmonary eosinophilia and were less effective in preventing influenza mediated impairment of lung function.^11^ Similar to these studies, adoptive transfer of *in vitro* generated IL‐17 producing CD8^+^ T cells can also protect recipient mice against influenza.^12^ Thus, regardless of the type of cytokines being produced, type 1, 2 and 17 CD8^+^ T cells are all capable of mediating protection against influenza, albeit to different degrees.^10^, ^11^, ^12^ Hamada *et al*.^13^ have confirmed the findings of these earlier studies and have shown that the CD8^+^T cells mediate protection against influenza via multilayered and redundant mechanisms. Therefore, type 1 biased immunity is not an absolute requirement for an effective vaccine against influenza. Other types of immunity, such as type 2 and 17, should also be considered. Indeed, an experimental influenza virus matrix protein‐based liposome vaccine that favours Th2 responses was shown to be protective in mice.^14^ In this study, CD4^+^ T cell vs. CD8^+^ T cell depletion of vaccinated mice showed that CD4^+^ T cells play a dominant yet not an obligatory role in mediating protection with no appreciable contribution of CD8^+^ T cells for survival.^14^ Collectively, these studies demonstrate that redundant protective mechanisms can participate to confer protection following influenza virus challenge, which includes several cell types and type 2 immunity.

## Role of group 2 innate lymphoid cells during influenza virus infection

Group 2 innate lymphoid cells were initially identified as a non‐B, non‐T cell population producing Th2 cytokine IL‐13 in mice. Due to the absence of antigen‐specific receptors, ILC2 are considered as an innate counterpart to Th2 cells.^15^ Detailed characterisation revealed that ILC2 are a lineage negative, CD45^+^ suppressor of tumorigenicity 2 (ST2)/IL‐33 receptor (IL‐33R)^+^ IL‐17 receptor B (IL‐17RB)^+^ population with variable expression of CD127, stem cell antigen‐1 and receptor tyrosine kinase (c‐kit).^16^, ^17^ Also, other receptors and ligands such as killer cell lectin like receptor G1 (KLRG1), inducible T cell co‐stimulator (ICOS) and IL‐18 receptor alpha chain (IL‐18RA) expressed on ILC2 are used to identify ILC2 subpopulations.^18^ In addition, ILC2 exhibit tissue‐specific phenotypes and different functions. For example, gut ILC2 mainly express KLRG1 and IL‐17RB, whereas lung ILC2 express ST2 and IL‐18RA.^19^ ILC2 development is driven by several transcription factors, namely, GATA binding protein 3 (GATA3), retinoic acid‐related orphan receptor α (RORα)^20^ and T‐cell specific high mobility group box transcription factor (TCF‐1).^21^ In the absence of antigen receptors, ILC2 are directly activated by epithelia‐derived alarmins. Activation by alarmins IL‐25, IL‐33 or thymic stromal lymphopoietin protein (TSLP) induces either ‘natural ILC2’ or ‘inflammatory ILC2’ functions. Natural ILC2 reside in the lungs and are induced by IL‐33 to perform homeostatic functions such as tissue remodelling, metabolism and epithelial repair.^22^ On the other hand, inflammatory ILC2 reside in the lymphoid tissues and the small intestine at steady‐state and migrate to lungs, spleen, liver and mesenteric lymph nodes upon stimulation by IL‐25.^23^ Lung ILC2 are a major source of type 2 cytokines IL‐5 and IL‐13 and are consequently heavily studied in the context of allergic lung inflammation and immunity against helminths or parasitic infections, which are diseases with type 2‐dominant immune response.^24^ In response to inhaled antigens, ILC2 contribute to the induction of Th2‐driven lung inflammation. Enhanced ILC2 levels and acute inflammation associated with increased production of type 2 cytokines IL‐4 and IL‐13 in the lungs have been implicated in allergic asthma in both mice and humans.^25^

Lung ILC2 also play an important role in protection against infections of acute respiratory viruses, such as influenza virus. Specifically, it has been shown that ILC2‐derived amphiregulin is crucial for improved lung function and repair following infection with the influenza virus strain A/PR/8/34 (PR8).^26^ ILC2 are also considered to be the major cells responsible for producing IL‐5 and IL‐13 during the late phase of influenza virus infection.^27^ Particularly, the production of IL‐5 by ILC2 during influenza virus infection is critical for the accumulation of eosinophils in the respiratory mucosae of mice, especially during the recovery phase of infection.^28^ We have previously published that these eosinophil recruitments may contribute to the recovery of influenza virus infected mice.^3^ In that study, we observed that the absence of IFN‐γ promoted the survival of influenza virus infected mice in an IL‐5 and ILC2 dependent manner. Notably, the increased resistance of IFN‐γ deficient mice was not associated with enhanced viral clearance but was correlated with reduced immunopathology and enhanced IL‐5 expressing ILC2 activity. In agreement with other studies,^29^, ^30^ we demonstrated that IFN‐γ effectively suppressed the function of lung ILC2. These data suggested that the increased production of IL‐5 by ILC2 in the absence of IFN‐γ was crucial to maintain tissue repair post‐infection and improve the survival of mice following CA04 infection.^3^ However, deficiency in signalling via type I interferon receptor (IFNAR1) led to increased activation of lung ILC2, susceptibility to i.n. infection with H1N1 influenza A virus /Puerto Rico/8/1934 (PR8) and infection‐associated type 2 immunopathology in mice in comparison to wild‐type C57BL/6 counterparts.^29^, ^30^ Differences between type I and type II interferons on ILC2 were further probed and indicated that type I interferons directly and negatively regulated ILC2 in interferon‐stimulated gene transactivation factor 3‐dependent manner, resulting in altered cytokine production, cell proliferation and increased cell death, whereas IFN‐γ and IL‐27 suppressed ILC2 function dependent on the signal transducer and activator of transcription 1.^29^, ^30^ In addition, contribution of ILC2 to protection against influenza virus infection was shown in a strain‐specific manner. The PR8 virus exhibited significantly increased virulence compared with the 2009 pandemic strain (CA04).^3^ Thus, PR8 used by Duerr *et al*.^29^ may overcome ILC2‐mediated protection.

Influenza‐associated morbidity and mortality disproportionately affects older adults. The increased susceptibility of elderly to severe influenza is partially attributed to age‐associated functional changes in ILC2. It was recently shown that lung ILC2 responses in aged mice (19–24 months old) were numerically and functionally compromised during influenza virus infection.^31^ Transfer of activated young ILC2 can rescue aged mice from influenza‐associated mortality. However, the protective mechanism of ILC2 was independent of viral clearance. The observed protection was associated with reduced lung pathology; a finding that is consistent with the literature that ILC2 promote survival primarily by alleviating airway inflammation.^31^

## Role of ILC2 in promoting adaptive memory responses

As discussed above, most of what we know about the role of ILC2 in influenza has been studied in a primary influenza virus challenge model and limited attention has been devoted to the contribution of ILC2 in establishing protective memory response following vaccination or sublethal influenza virus infection. ILC2 have been shown to impact both innate and adaptive components of the immune system in various disease models.^32^ Intriguingly, ILC2 appear to play a role in the induction of adaptive memory responses.

IL‐33, a ILC2‐activating cytokine, and ILC2 are induced during primary influenza virus infection prior to recruitment of B and T cells. This allows IL‐33 activated ILC2 to influence and regulate the ensuing adaptive immunity. For example, lung ILC2 are a crucial link between allergen‐induced epithelial cytokine production and initiating cell‐mediated allergic lung inflammation, promoting both innate and adaptive immune responses.^33^ ILC2 have been also demonstrated to interact with other innate immune cells, specifically, antigen presenting cells. Upon allergen exposure, the production of ILC2‐derived IL‐13 is critical for inducing migration of DCs to mediate activation and differentiation of CD4^+^ T cells into Th2 memory cells.^32^, ^34^ ILC2 can also interact with regulatory T cells (Tregs), which can either exacerbate or control inflammatory disease progression. For example, ILC2‐induced IL‐4 has been shown to promote food allergy by blocking Treg function.^35^ In contrast, the activation of Tregs by ILC2‐derived IL‐9 leads to the resolution of inflammation.^36^ It has also been demonstrated that, under some conditions, ILC2 can directly present antigens via major histocompatibility complex (MHC)‐II molecules to potentiate differentiation of Th2 cells, while suppressing Th1 responses.^37^ Through the use of two distinct ILC2‐deficient mouse strains and an adoptive cell transfer system, it was shown that MHC‐II mediated cooperation between ILC2 and CD4^+^ T cells are required for effective adaptive type 2 anti‐helminth immunity.^38^ It should be noted that MHC‐II expression on ILC2 are low and transient, and therefore their contribution to antigen presentation relative to other conventional APCs may be insignificant. However, the authors did demonstrate that ILC2 can compensate for the loss of DCs in supporting proliferation of T cell receptor (TCR) transgenic T cells *in vitro*.

In addition to T cell immunity, ILC2 can also impact humoral immune responses. IL‐5 produced by ILC2 has been demonstrated to be crucial for the maintenance and activation of B cells for antibody production.^16^, ^39^ Furthermore, ILC2 can also promote follicular B cells and support production of IgM, IgG1, IgA and IgE antibodies in mice.^40^ While above investigations involved allergens and parasites, ILC2 may exhibit similar immunological mechanisms during influenza vaccination to regulate the immune memory response to vaccine antigens. More recent studies have studied the role of IL‐5 produced by ILC2 in promoting humoral immunity. In particular, upon nematode infection of mice, IL‐5 production by ILC2 has been shown to activate pleural native B cells for IgM production and cause differentiation of follicular B cells.^39^ Similarly, following the induction of airway inflammation, ILC2 were shown to produce antigen‐specific IgM antibodies in mice. Specifically, using IL‐5 deficient mice authors showed that ILC2‐derived IL‐5 promotes IgM as opposed to IgG1 antibody production.^40^ IgM antibody production by ILC2‐derived IL‐5 also exhibits protective effects against development of atherosclerosis in mice.^41^

It should also be noted that ILC2 have been shown to acquire some of the characteristics of immune memory cells upon initial activation, such as being a faster and/or stronger responder to secondary challenges.^42^ Unlike naïve ILC2, ‘trained’ counterparts exhibit sustained survival up to months and expression of IL‐25R and CD25 markers.^22^, ^43^ Consequently, ILC2 with the trained immunity phenotype can produce higher levels of IL‐4 and IL‐13 compared to naïve ILC2 following subsequent reactivation.^43^ However, due to the lack of rearranged antigen receptors, trained ILC2‐mediated memory is non‐antigen specific, similar to cytokine‐induced memory‐like NK cells.^43^ Thus, the role of ‘trained’ ILC2 in vaccine mediated protection is difficult to elucidate, but remains an important area of future investigation.

## Targeting lung ILC2 during i.n. vaccination

Group 2 innate lymphoid cells express a number of cytokine receptors that are a potential target to modulate ILC2 functions to favourably influence vaccine efficacy. For example, i.n. vaccination with fowlpoxviral (FPV) vector‐based HIV vaccine stimulates expansion of IL‐33R^+^ ILC2 in the lung mucosae,^44^ and this response correlates strongly with the induction of high avidity cytotoxic CD8^+^ T cell responses in mice and macaques.^45^, ^46^ Although how viral vector‐based HIV vaccines activate ILC2 remain elusive, our data demonstrates that the choice of viral vector and the route of vaccination crucially impact the ILC2 response. Different viral vectors, containing same HIV antigens have different capacity to stimulate IL‐13 expression by IL‐33R^+^ ILC2 in the lung.^44^ In particular, FPV‐induced low levels of IL‐13 correlated with enhanced lung cDC recruitment^44^ and high avidity antigen‐specific CD8^+^ T cells that are thought to be important for the vaccine efficacy.^47^ In contrast, induction of high IL‐13^+^ ILC2 by other vectors such as recombinant vaccinia virus (rVV) correlated with the preferential recruitment of lung cross‐presenting DCs.^48^ These cross‐presenting DCs have been associated with low vaccine efficacy against HIV in mice due to the induction of low avidity CD8^+^ T cells.^47^ These studies demonstrate that viral vector‐based vaccines can be used to shape lung ILC2 functions to elicit favourable vaccine‐induced responses. Indeed, recent studies have shown that ILC2 exhibit functional plasticity and are capable of secreting type 1 cytokine IFN‐γ in response to IL‐12 stimulation.^49^, ^50^ We have also recently reported that ILC2 functions can be modulated by the presence of IFN‐γ during influenza virus infection.^3^ Plasticity of ILC2 functions enables them to exert potent immunomodulatory effects through the secretion of various cytokines including both type 1 (IFN‐γ) and type 2 (IL‐4 and IL‐13) cytokines.^49^ Thus, ILC2 represent an attractive target for mucosal vaccination. ILC2 functions can be manipulated to induce desired immune responses, either type 1 or type 2 cytokines, that are appropriate for a given vaccine formulation.

In addition to viral‐vector based vaccination, exogenous recombinant IL‐33 (rIL‐33) inoculation has also been shown to enhance antiviral immunity, presumably mediated by lung ILC2. Repeated i.n. administration of rIL‐33 for 5 days prior to PR8 infection improved survival of mice and decreased viral titers compared to mice that did not receive exogenous IL‐33.^51^ This i.n. administration of IL‐33 increased ILC2, DC and eosinophil recruitment into the lungs, promoting proinflammatory cytokine secretion and cytotoxic T cell responses.^51^

**Figure 1:**
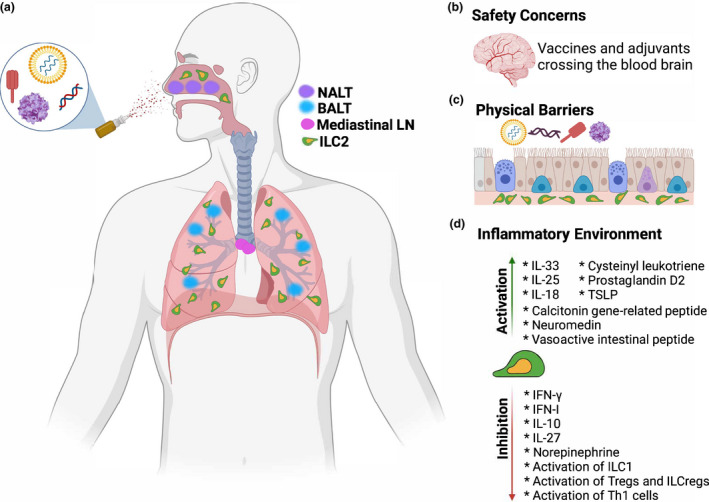
Challenges that need to be overcome to develop intranasal vaccines that can potently activate group 2 innate lymphoid cells (ILC2) function in the respiratory mucosa and the lungs. **(a)** ILC2 in the respiratory system appear to predominantly reside in the lungs at steady state although under certain inflammatory/infectious settings (e.g. chronic rhinosinusitis) ILC2 can be recruited to the vicinity of upper respiratory tract such as the nasal cavity and nasal associated lymphoid tissue (NALT). Antibodies (e.g. IgA) and/or tissue‐resident memory B and T cells at the upper respiratory tract are likely required to elicit sterilising immunity and protective immunity at the lower respiratory tract is required to mitigate disease pathology resulting from respiratory virus infection. Consequently, an intranasal vaccine will likely need to deposit/express antigen in ILC2 ‘hotspots’ such as the nasal cavity and the lungs and trigger inflammation in neighbouring lymphoid tissues such as the NALT, bronchus‐associated lymphoid tissue (BALT) and mediastinal lymph nodes (LN). This will be important to maximise the activity of ILC2 and their capacity to prime adaptive immunity in the upper and lower respiratory tract. Apart from the requirement to deposit/express vaccine antigens at relevant hotspots **(a)**, there are several other challenges. **(b)** There are inherent risks if the vaccine and/or adjuvants cross the blood brain barrier, which could trigger inflammation in the brain. **(c)** Numerous cell types in the epithelium form a formidable physical barrier to capture pathogens and vaccine antigens limiting the access of such antigenic components to ILC2. Despite this barrier preventing antigen access to ILC2, other antigen presenting cells such as dendritic cells can capture antigens to promote inflammation and the epithelial cells can secrete alarmins such as IL‐33 to where ILC2 reside following exposure of antigenic components. Consequently, ILC2 activation could still ensue in this context. **(d)** There are various cell‐associated and soluble factors that can activate or inhibit ILC2 function.^56^ It is unlikely that an intranasal vaccine will be able to exclusively promote the development of activating factors compared to inhibitory factors of ILC2, but it is important for the vaccine to bias the elicitation of activating factors of ILC2 especially those that can help these cells function as antigen presenting cells in the upper and lower respiratory tract. The figure was constructed using BioRender.com.

In addition, we have recently demonstrated that co‐administering recombinant IL‐33 (rIL‐33) with inactivated influenza vaccine (IIV) intranasally significantly enhances vaccine efficacy not only against vaccine matched homologous H1N1 but also against antigenically drifted heterologous H1N1 influenza virus infection in mice.^52^ Investigating underlying mechanisms also revealed that rIL‐33 adjuvanted IIV potentiates the observed cross‐protection by early activation of lung ILC2 resulting in robust humoral immunity, specifically IgA antibodies in the lung mucosae.^52^ There are several challenges that need to be overcome to develop an effective intranasal vaccine to harness the desirable functions of ILC2 in potentiating protective immune responses against respiratory virus infections, which is described in Figure 1. However, our studies and the findings of others suggest that targeting ILC2 in the lungs and respiratory mucosa is feasible and likely will be highly effective in enhancing the immunogenicity and protective capacity of intranasally delivered vaccine antigens against respiratory virus infections.

## Potential detrimental consequences to an ILC2 targeted vaccination approach

Influenza virus like other respiratory viruses such as rhinovirus and respiratory syncytial virus (RSV) are the most common triggers for allergic asthma exacerbations, and come at a burden to patients, healthcare system and the economy.^53^, ^54^ These viral infections occur mainly in the respiratory tract and subsequent expansion of ILC2 may contribute to the development and/or exacerbation of asthma.^55^ Specifically, influenza virus boosts the host’s allergen response and causes significant morbidity and mortality in patients with asthma by rapidly inducing pulmonary lung inflammation and airway hyperresponsiveness (AHR) through the activation of ILC2.^54^ Enhanced ILC2 and subsequent type 2 cytokine overactivity in the lung after influenza virus infection may promote AHR, regardless of a patients’ asthma history.^27^, ^54^ Specifically, excessive IL‐33 production during respiratory viral infections has been shown to impact antiviral immune responses and exacerbate virus‐induced asthma in an animal model.^56^ Similarly, targeting ILC2 during i.n. influenza vaccination may skew the recall anti‐influenza responses towards type 2 immunity and consequently may worsen influenza‐induced asthma exacerbation upon future infection via upregulation of type 2 cytokines. These concerns warrant future investigation. Nevertheless, our promising findings demonstrate that rIL‐33 adjuvanted IIV vaccine, despite triggering lung ILC2, causes no cytotoxicity and damage in vaccinated murine lungs.^52^

It should be noted that type 2 cytokines also possess antiviral properties. For example, IL‐5 is a well‐known chemoattractant for eosinophils.^55^ In response to infection, eosinophils from healthy individuals have been shown to capture and inactivate influenza virus to promote viral clearance.^57^ In a mouse model of asthma, pandemic H1N1 influenza virus infection caused increased pulmonary eosinophilia.^58^ Interestingly, asthmatic mice are resistant to influenza virus infection and the protection was attributed to immunoregulatory functions of eosinophils. Eosinophils in allergen challenged mice promotes antigen presentation and activation of antigen specific CD8^+^ T cells.^59^ In addition to immunoregulatory functions, eosinophils can also exert direct antiviral responses. Specifically, *in vitro,* human eosinophils were shown to reduce infectivity of RSV and parainfluenza viruses by secreting eosinophil derived neurotoxin (EDN).^60^ Additionally, *in vivo*, eosinophils triggered by allergen exposure can promote viral clearance following parainfluenza virus infection in guinea pigs.^61^ Adoptive transfer of splenic eosinophils from hypereosinophilic mice also accelerated RSV clearance in wild type mouse lungs post infection in a MyD88 dependent manner.^62^ Furthermore, using a replication competent close relative of RSV (PVM), Percopo *et al*.^63^ also showed that hypereosinophilic mice promote enhanced survival and viral clearance in mouse lungs.

In addition to type 2 cytokine responses, activated ILC2 during influenza virus infection also secrete amphiregulin.^64^, ^65^ Amphiregulin production by ILC2 plays a beneficial role during the recovery phase of infection of virus‐induced asthma exacerbation, and the depletion of ILC2 and the subsequent decrease of amphiregulin has been shown to result in diminished lung function and loss of airway epithelial integrity in mice.^66^

## Conclusion

Lung ILC2 are key players that link innate and adaptive components of the mucosal immune system. Cytokines produced by activated lung ILC2 during influenza infection have been shown to play dual roles. While early ILC2‐derived cytokines can protect against influenza, ILC2 accumulation and enhanced type 2 inflammation has been deemed to exacerbate infection and even promote influenza‐induced asthma. However, capitalising on the protective roles of ILC2‐induced Th2 immunity have given birth to new ammunition to aid the development of new vaccination strategies against viral pathogens. As a proof of concept, our data demonstrates that activation of lung ILC2 at the time of i.n. vaccination can vastly improve immunogenicity and cross‐protective efficacy of inactivated influenza vaccines against heterologous influenza infection. It will be important to determine whether the vaccine enhancing effects of lung ILC2 is specific to influenza vaccine, or it is general property of ILC2 that can be applied to other vaccines. It is clear that ILC2 activation can be protective following respiratory virus infection and consequently an intranasal vaccination strategy that will activate ILC2 in the right context as we have described will likely be highly effective in conferring protection. Further research is required to establish the potential utility of lung ILC2 in modulating host vaccine responsiveness. In particular, investigating not only the beneficial but also adverse contributions of lung ILC2 during pulmonary viral infections will be crucial as such knowledge will allow fine‐tuning of ILC2 functions in order to enhance the efficacy of mucosal vaccines.

## Conflict of interest

The authors declare no conflict of interest. The funders had no role in the writing of the manuscript or in the decision to publish.

## Author Contributions

**Clare M Williams:** Writing – original draft; Writing – review & editing. **Sreeja Roy:** Writing – original draft; Writing – review & editing. **Wei Sun:** Writing – review & editing. **Andrea M Furuya:** Writing – original draft; Writing – review & editing. **Danushka K Wijesundara:** Conceptualization; Writing – original draft. **Yoichi Furuya:** Conceptualization; Funding acquisition; Writing – review & editing.
